# Disease modeling of ADAMTS9-related nephropathy using kidney organoids reveals its roles in tubular cells and podocytes

**DOI:** 10.3389/fmed.2023.1089159

**Published:** 2023-03-23

**Authors:** Seyoung Yu, Yo Jun Choi, John Hoon Rim, Hye-Youn Kim, Nasim Bekheirnia, Sarah Jane Swartz, Hongzheng Dai, Shen Linda Gu, Soyeon Lee, Ryuichi Nishinakamura, Friedhelm Hildebrandt, Mir Reza Bekheirnia, Heon Yung Gee

**Affiliations:** ^1^Department of Pharmacology, Graduate School of Medical Science, Brain Korea 21 Project, Yonsei University College of Medicine, Seoul, Republic of Korea; ^2^Department of Laboratory Medicine, Yonsei University College of Medicine, Seoul, Republic of Korea; ^3^Department of Pediatrics, Division of Pediatric Nephrology, Baylor College of Medicine, Houston, TX, United States; ^4^Department of Molecular and Human Genetics, Baylor College of Medicine/Baylor Genetics, Houston, TX, United States; ^5^Department of Kidney Development, Institute of Molecular Embryology and Genetics, Kumamoto University, Kumamoto, Japan; ^6^Department of Medicine, Division of Nephrology, Boston Children's Hospital, Boston, MA, United States; ^7^Department of Molecular and Human Genetics, Baylor College of Medicine, Houston, TX, United States

**Keywords:** Nephronophthisis-related ciliopathy, ADAMTS9, kidney organoid, single-cell RNA sequencing, podocytes

## Abstract

**Introduction:**

Mutations in *ADAMTS9* cause nephronophthisis-related ciliopathies (NPHP-RC), which are characterized by multiple developmental defects and kidney diseases. Patients with NPHP-RC usually have normal glomeruli and negligible or no proteinuria. Herein, we identified novel compound-heterozygous *ADAMTS9* variants in two siblings with NPHP-RC who had glomerular manifestations, including proteinuria.

**Methods:**

To investigate whether ADAMTS9 dysfunction causes NPHP and glomerulopathy, we differentiated ADAMTS9 knockout human induced pluripotent stem cells (hiPSCs) into kidney organoids. Single-cell RNA sequencing was utilized to elucidate the gene expression profiles from the *ADAMTS9* knockout kidney organoids.

**Results:**

*ADAMTS9* knockout had no effect on nephron differentiation; however, it reduced the number of primary cilia, thereby recapitulating renal ciliopathy. Single-cell transcriptomics revealed that podocyte clusters express the highest levels of ADAMTS9, followed by the proximal tubules. Loss of ADAMTS9 increased the activity of multiple signaling pathways, including the Wnt/PCP signaling pathway, in podocyte clusters.

**Conclusions:**

Mutations in *ADMATS9* cause a glomerulotubular nephropathy in kidney and our study provides insights into the functional roles of ADMATS9 in glomeruli and tubules.

## 1. Introduction

Nephronophthisis (NPHP) is a clinically and genetically heterogeneous disorder associated with the dysplasia and degeneration of the kidneys. In some patients with NPHP, liver, retina, and central nervous system are also affected (1). NPHP is characterized by massive interstitial fibrosis with abnormal thickness of the tubular basement membranes, and in later stages, the formation of cysts that are mainly distributed at the corticomedullary junction (1). A common set of genes are reportedly associated with NPHP development and the formation of primary cilia, sensory organelles that respond to chemical and mechanical stimuli and transmit external signals to the cell’s interior (2). In NPHP-related ciliopathies (NPHP-RC), glomeruli are mostly normal, although secondary sclerosis is often observed at advanced stages and proteinuria is absent or minimal (3). Recently, several genes have been identified with specific roles in regulating the glomerular filtration barrier function and primary cilium homeostasis. TTC21B, which encodes for IFT139, is associated with disorders of both glomerular and tubulointerstitial compartments (4).

Recessive *ADAMTS9* mutations have been associated with the development of NPHP-RC (5, 6). ADAMTS9 belongs to a disintegrin and metalloprotease with thrombospondin motifs (ADAMTS) family and is a secreted metalloprotease found in both mammals and invertebrates (7). ADAMTSs are extracellular multi-domain enzymes that regulate the extracellular matrix (ECM) and facilitate cell migration by degrading proteoglycans and collagen (8–10). ADAMTS9 is localized near the basal body of primary cilia and is involved in regulating ciliogenesis (5, 11). ADAMTS9 dysfunction results in the shortening or loss of the primary cilium. The reported renal manifestations associated with mutations in ADAMTS9 include corticomedullary cysts, immature glomeruli, and proteinuria (5).

Proteinuria arises due to a dysfunctional glomerular filtration barrier, which is composed of three cell layers: glomerular endothelial cells, glomerular basement membrane (GBM), and glomerular visceral epithelial cells (podocytes). Proteinuria is a characteristic of focal segmental glomerulosclerosis (FSGS), a renal histological lesion with various causes associated with podocyte injury (12). It is now widely accepted that the podocyte slit diaphragm, a specialized cell junction, plays a critical role in preventing the leakage of plasma proteins into primary urine, and the dysfunction of the slit diaphragm is involved in the development of proteinuria in several glomerular diseases (13).

To investigate the effects of ADAMTS9 dysfunction, knockout and knockdown strategies have been used in cell lines and model organisms including Caenorhabditis elegans and mice (5, 11, 14–18). However, interspecies variations and the inability to recapitulate the phenotype exhibited by the affected individuals are causes of concern while using these experimental models (19). With the identification of nephron progenitors that could give rise to podocytes and proximal tubules, it has now become easy to generate three-dimensional kidney tissues from human induced pluripotent stem cells (hiPSCs) (20). The establishment of kidney organoids has facilitated the study of human genetic diseases. Single-cell RNA sequencing (scRNA-seq) has been widely used to identify cell type-specific regulatory relationships among genes (21). This is particularly useful for resolving cellular heterogeneity in human iPSC-derived functional cell types.

Herein, we identified compound heterozygous variants of ADAMTS9 in patients with NPHP and nephrotic-range proteinuria. To investigate the role of ADAMTS9 in primary cilia formation and the pathogenicity of the identified ADAMTS9 variants, we generated kidney organoids from patient specific hiPSCs. Single-cell transcriptomic analysis was performed to elucidate the role of ADAMTS9 in podocytes. Finally, to confirm the role of ADAMTS9 in regulating the localization of podocyte junction proteins nephrin and podocin, immunofluorescence staining was performed.

## 2. Materials and methods

### 2.1. Exome sequencing

Trio exome sequencing was performed at Baylor Genetics as previously described (1). This analysis of patient data and clinical genomics data was approved by the Institutional Review Board at Baylor College of Medicine. For target enrichment/exome capture, the pre-capture library was enriched by hybridizing with biotin-labeled VCRome 2.1 in solution exome probes. For massive parallel sequencing, the post-capture DNA library was subjected to sequence analysis on the Illumina HiSeq platform to obtain 100 bp paired-end reads. Sequence reads were mapped against the human reference genome (NCBI build 37/hg19) using the Dragen software (Illumina). Variant calling, annotation, and filtering were performed using an in-house bioinformatics pipeline as described previously (1).

### 2.2. Plasmid construction and site-directed mutagenesis

Human *ADAMTS9* cDNA was purchased from OriGene Technologies and subcloned into the pENTR-D-TOPO vector (Invitrogen). Expression vectors were created using LR Clonase (Invitrogen), following the manufacturer’s instructions. Clones reflecting *ADAMTS9* mutations identified in individuals with NPHP-RC were introduced into cDNA constructs in the pENTR-D-TOPO vector using the QuikChange II XL site-directed mutagenesis kit (Agilent Technologies).

### 2.3. Cell culture and transfection

Human embryonic kidney 293 (HEK293, CRL-1573) and hTERT retinal pigment epithelial-1 (RPE1, CRL-4000) cells were obtained from ATCC. HEK293 cells were cultured in high glucose DMEM with 10% FBS and penicillin (50 IU/mL)/streptomycin (50 μg/mL), while RPE1 cells were cultured in DMEM/F12 with 10% FBS and penicillin (50 IU/mL)/streptomycin (50 μg/mL). HEK293 cells were transfected with plasmids containing C-terminal V5-tagged wild type (WT) or mutant *ADAMTS9* using the Lipofectamine PLUS reagent (Invitrogen) according to the manufacturer’s instructions. RPE1 cells were transfected with cDNA constructs containing C-terminal V5-tagged WT or mutant *ADAMTS9* using Lipofectamine 2000 (Invitrogen) according to the manufacturer’s instructions. To examine the pathogenicity of *ADAMTS9* mutations identified in this study, IMR90 (6.25*10^4^ cells) were transfected with cDNA constructs containing C-terminal V5-tagged WT or mutant *ADAMTS9* (10 μg) using electroporation (NEPA21, Nepa gene) according to the manufacturer’s instructions. Single iPSC colony was picked and analyzed for further differentiation steps.

### 2.4. Generation of ADAMTS9 knockout human induced pluripotent stem cells (hiPSCs)

To establish ADAMTS9 knockout iPSCs, Precision gRNA Synthesis Kit (Invitrogen, A29377) and TrueCut™ Cas9 Protein v2 (Invitrogen, A36496) were used. gRNAs targeting ADAMTS9 were synthesized according to the manufacturer’s instruction: gRNA1, 5’-AGCGATAAATCCGGCATTGG-3′; gRNA2, 5’-ACACGATTTCGTATTCGCTC-3′; gRNA3, 5’-TCCGTCGCGTTCTTTTAGGA-3′. Four clones were subsequently adapted to feeder-free conditions and were confirmed by sanger sequencing. Among them, two clones were able to differentiate into kidney organoids and these two clones were used for further studies. Two independent induction experiments were performed for each clone, and at least 16 organoids per clone were generated in each experiment. Four control organoids and three of each knockout organoids were sorted by FACS to confirm the embryonic nephron progenitor portions. After induction toward nephron progenitor cells, three organoids per clone in each experiment were subjected to serial sectioning for immunostaining and remaining organoids (9 control organoids and 10 knockout organoids) were used for scRNA-seq.

### 2.5. Generation of kidney organoids from hiPSCs

We followed the *in vitro* induction protocol of Taguchi et al. (2) for generating nephron progenitors that were used to produce kidney organoids. The human iPSC line WISCi004-A (IMR90), which is produced by the lentiviral reprograming of IRM90.4 fibroblasts, was obtained from WiCell. The serum-free hiPSC differentiation medium consisted of DMEM/F12 (Invitrogen) supplemented with 2% (v/v) B27 (without retinoic acid), 1% GlutaMAX, 1% (v/v) ITS, 1% (v/v) nonessential amino acids, 90 μM β-mercaptoethanol, and 1% (v/v) penicillin/streptomycin. Cells (10,000 per well) were plated in V-bottom 96-well low-cell-binding plates to form EBs in the presence of 10 μM Y27632, 1 ng/mL human activin A, 20 ng/mL human bFGF and 1 ng/mL BMP4. After 24 h (on day 1), the cell aggregates were transferred to the medium containing 10 μM CHIR in U-bottom 96-well low cell-binding plates. Subsequently, half of the culture medium was replaced with fresh medium containing 10 μM CHIR and 10 μM Y27632 on days 3 and 5. On day 7, culture medium was removed and fresh medium containing 10 ng/mL human activin A, 3 ng/mL human Bmp4, 3 μM CHIR, 0.1 μM RA, and 10 μM Y27632 was added. On day 10, the culture medium was removed and fresh medium containing 1 μM CHIR, 5 ng/ml human Fgf9, and 10 μM Y27632 was added. On day 13, the aggregated spheres were transferred to a 24-well transwell insert (Corning) and cultured in DMEM/F12 (Invitrogen) supplemented with 1% nonessential amino acids, 5 mM HEPES, 5% (v/v) knockout serum replacement (KSR), 1% GlutaMAX, 50 μM β-mercaptoethanol, and 1% (v/v) penicillin/streptomycin with 3 μM CHIR for the first 48 h. The medium was then replaced every other day with the medium without CHIR.

### 2.6. Flow cytometric analysis

We followed the protocol of Taguchi et al. (2). Spheres induced from iPSCs were dissociated and blocked with normal mouse serum for 10 min on ice. ITGA8, cell surface marker, staining was carried out in 1× HBSS containing 1% BSA and 0.035% NaHCO3 for 30 min on ice. Stained cells were analyzed using FACS Verse II (BD Biosciences). Data analyzes were performed with FACS Diva software. Quantification data were presented as mean ± SEM.

### 2.7. Sample preparation for single-cell RNA sequencing

On day 29 following transfer to the 24 well transwell insert, the spheres were dissociated using Accumax at 37°C for 20 min. After centrifugation, the supernatant was removed and cell washing buffer (1× Hank’s balanced salt solution [HBSS] containing 20% FBS, 50 ng/mL DNaseI, and 0.035% NaHCO_3_) was added. Single cell suspensions were prepared using a fresh plunger of a sterile 5 mL syringe. Cells were resuspended in 0.04% bovine serum albumin (BSA) in phosphate-buffered saline (PBS), and passed through a 40 μm cell strainer (Falcon; Cat# 352340). Cell number and viability were evaluated using the Countess automated cell counter.

### 2.8. Single-cell RNA sequencing analysis

*Preprocessing of 10 × Cellplex sequencing output.* Each organoid was dissociated into single cells that were multiplexed using the CellPlex Kit (10 × Genomics) and the Chromium Controller (10 x Genomics). The 3-plexed cells were pooled together and loaded onto 10 × chromium chips. The Chromium Single Cell 3′ Library & Gel Beads Kit v3.1 (10 × Genomics) was used to generate cDNA libraries, which were then sequenced using the Illumina HiSeq 3,000 platform. The Cellranger toolkit (version 6.1.2) was used to perform demultiplexing using the *cellranger multi* command for alignment to the pre-mRNA transcriptome, cell barcode partitioning, collapsing unique molecular identifier (UMI) to transcripts, and gene-level quantification. These experiments were performed twice, and the data from Cellranger were integrated for further analysis. Genes were filtered to include only those that were expressed in at least three cells. Cells were filtered to include only those cells expressing a minimum of 200 and a maximum of 6,000 genes. The percentage of reads mapped to mitochondrial genes was capped at 5%. For each line, we obtained more than 5,400 valid cells (> 200 expressed genes) after filtering, with the profiling of over 3,300 genes and total of 12,093 cells.

*Unsupervised clustering and dimensionality reduction.* Downstream analysis was performed using the Seurat R package (3), version 4.1. Data normalization (log1p[counts]), variable gene identification, and scaling (regularized negative binomial regression) were performed, and unwanted variations due to variations in nFeature_RNA and percent. Mito were subsequently regressed out (SCTransform, vars.to.regress = c[“nFeature_RNA,”“percent.mito”]). The samples were then integrated using the default settings (FindIntegrationAnchors, IntegrateData). Following data integration, the combined dataset was rescaled (ScaleData, vars.to.regress = c[“nFeature_RNA,”“percent.mito”]). Dimensionality reduction was performed using principal component analysis (RunPCA) of highly variable genes. The ElbowPlot function was used to distinguish principal components (PCs) for further analysis. Fifteen components were sufficient to capture variance. We identified molecularly distinct clusters using default parameters (FindNeighbors, FindClusters), with a resolution of 0.5. We computed and embedded the data in 2-D space using uniform manifold approximation and projection (UMAP) in PC space for visualization (RunUMAP, 16 PCs).

*Assignment of cell identity*. Cluster-enriched or marker genes were identified using Wilcoxon’s rank sum test (FindAllMarkers) for differentially expressed genes in one cluster cell versus all other cells, filtering for genes with a log2FC greater than 0.25. Cluster identities were assigned by comparing data-driven genes with a list of literature-curated genes in mature kidney cell types. For GSEA, the enrichment of all genes was determined using default parameters, except that the log2FC threshold was set to 0 (FindMarkers). GSEA was carried out on positive- and negative-enriched genes separately. Leading-edge genes were extracted for each pathway, and hierarchical clustering (hclust) was performed on the pathways based on overlap of leading-edge genes.

*Differential gene expression analysis*. For evaluating differential expression between the control and *ADAMTS9* knockout samples, we performed pair-wise differential expression analysis (FindMarkers) with default parameters.

*Trajectory analysis*. Pseudotime trajectory analysis on nephrons was performed using Monocle3 (4–6). The intersection of the top 100 genes with the greatest absolute fold-change for each nephron cluster was selected for this analysis.

*Cell-to-cell communication analysis.* CellphoneDB is a Python-based computational analysis tool developed by Roser Vento-Tormo et al. (7) that can analyze cell–cell communication at the molecular level. Interaction pairs of PDGF, NOTCH, Wnt, TGF-β, VEGF, and BMP family proteins were selected to evaluate the relationship among cell types. The *plot_cpdb* function was used in the ktplots package^1^ to visualize the dot-plot.

### 2.9. Immunoblotting and immunofluorescence

Anti-ADAMTS9 (Novus, NBP1-82916), anti-β-actin (Abcam, ab6276), anti-acetylated-α-tubulin (Cell Signaling Technology, 5335S), and anti-V5 (Cell Signaling Technology, 80076S) antibodies were used. Alexa Fluor 488- and Alexa Fluor 594-conjugated secondary antibodies and 4′,6-diamidino-2-phenylindole dihydrochloride (DAPI) were obtained from Invitrogen. Horseradish peroxidase (HRP)-labeled secondary antibodies were purchased from Santa Cruz Biotechnology. At 24 h after transfection, the cells were switched to serum-free medium. After 60 h, cells were harvested and lysed. Serum-free spent medium was collected separately and concentrated using Amicon Ultra-4 (Millipore). Protein samples were separated using sodium dodecyl sulfate-polyacrylamide gel electrophoresis. The separated proteins were transferred to nitrocellulose membranes and blotted with the indicated primary antibodies. Confocal images were obtained using the Carl Zeiss LSM780 microscope. Image processing and analysis were performed using the ZEN software. For immunofluorescence, RPE1 cells were seeded at a low density, grown to confluence, and then serum-starved for 48 h to induce primary cilia. ADAMTS9 immunoperoxidase images were obtained from the Human Protein Atlas with the original source available at the following link: (https://www.proteinatlas.org/ENSG00000163638-ADAMTS9/tissue/kidney).

### 2.10. Electron microscopy

The kidney organoids were fixed in 2.5% glutaraldehyde, 1.25% paraformaldehyde, and 0.03% picric acid in 0.1 M sodium cacodylate buffer (pH 7.4) overnight at 4°C. They were then washed with 0.1 M phosphate buffer, postfixed with 1% osmium tetroxide dissolved in 0.1 M PBS for 2 h, dehydrated in ascending gradual series (50–100%) of ethanol, and propylene oxide was used for infiltration. Samples were embedded using the Poly/Bed 812 Kit (Polysciences) according to manufacturer’s instructions. After pure fresh resin embedding and polymerization in a 65°C oven (TD-700; DOSAKA, Kyoto, Japan) for 24 h, sections of approximately 200–250 nm thickness were cut and stained with toluidine blue for light microscopy. Sections of 70 nm thickness were double stained with 6% uranyl acetate (22,400; EMS) for 20 min and lead citrate (Fisher) for 10 min for contrast staining. The sections were cut using a Leica EM UC-7 with a diamond knife (Diatome) and transferred onto copper and nickel grids. All the sections were observed by transmission electron microscopy (TEM; JEM-1011, and JEOL) at an acceleration voltage of 80 kV.

### 2.11. Statistics

All data are presented as mean ± standard deviation of the mean obtained from the indicated number of experiments. Statistical analysis of continuous data was performed using the two-tailed Student’s *t-*test. A *p*-value <0.05 was considered statistically significant.

## 3. Results

### 3.1. Identification of recessive *ADAMTS9* variants in a family with multiple congenital anomalies

**Figure 1:**
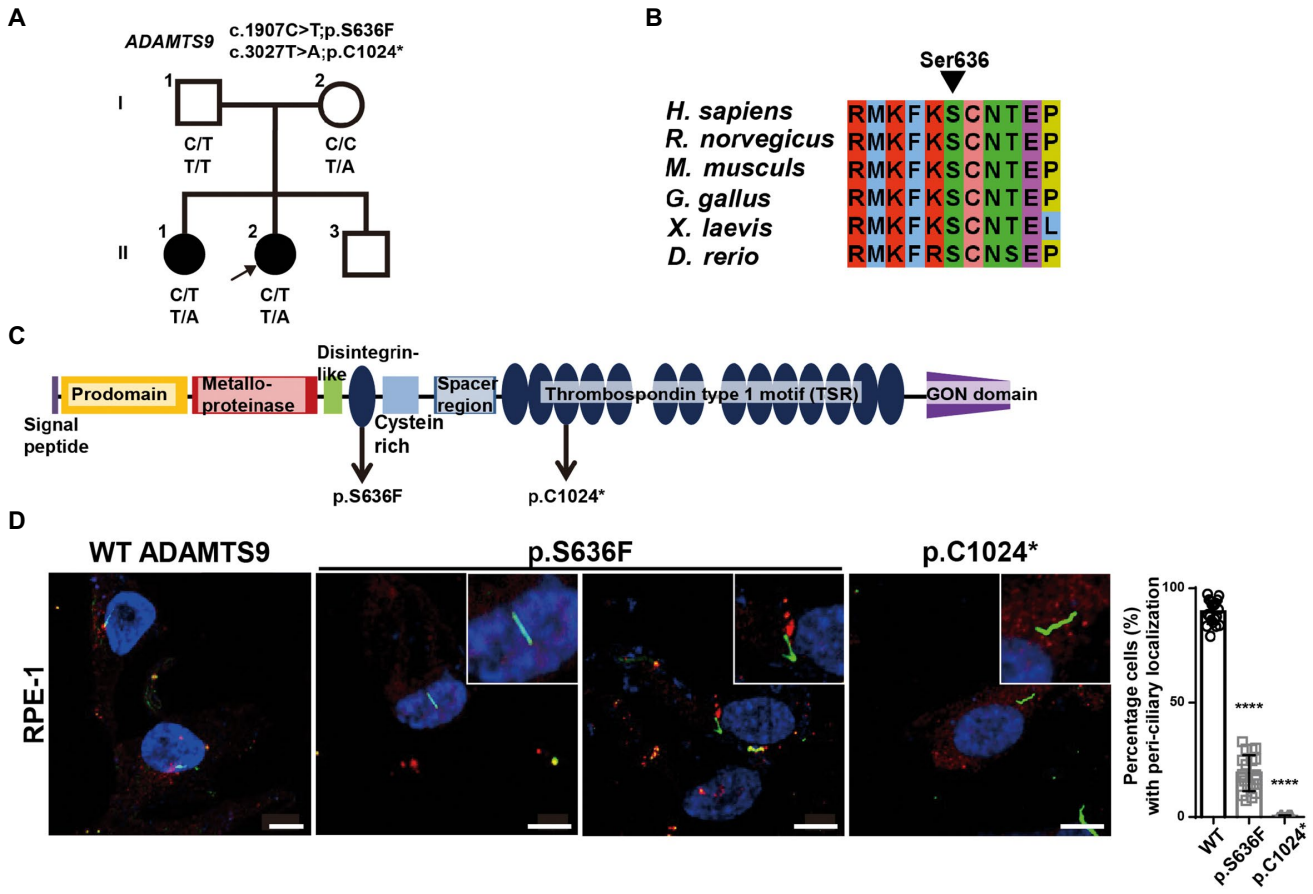
*ADAMTS9* variants identified in patients with NPHP. **(A)** Pedigree of the family with NPHP. **(B)** The newly identified *ADAMTS9* mutations altered the evolutionary conserved amino acid residues in the ADAMTS9 protein. **(C)** Domain structure of wildtype (WT) ADAMTS9 and NPHP-associated variants. **(D)** Localization of overexpressed ADAMTS9 in RPE1 cells. Cells were transfected with V5 tagged WT and mutant ADAMTS9 cDNA constructs, serum starved for 48 h, and stained with anti-Ac-α-tubulin and anti-V5 antibodies. Quantification is shown in the right panel. Scale bar, 10 μm. ^****^p < 0.0001.

The proband was an 8-year-old Caucasian female at the time of the first genetic evaluation, born at 39 weeks of gestation *via* natural delivery, and had abnormal prenatal ultrasound findings, including hydrocephaly. Postnatally, multiple congenital anomalies were identified, including bilateral iris and retinal coloboma, absence or agenesis of the corpus callosum, bicornuate uterus, anorectal anomaly requiring anorectoplasty, and a tied tongue that was surgically repaired. Growth parameters revealed a normal FOC of 50 cm at the 20th percentile. However, her stature was 111 cm, which is less than the 3rd percentile (50th percentile of a 5.5-year-old female). Her weight was 19.6 kg, approximately at the 3rd percentile. Other physical findings included dysmorphic ears with simplified helices and attached earlobes. Renal manifestations included proteinuria in the nephrotic range, and renal ultrasound revealed bilateral abnormal echogenic kidneys with decreased corticomedullary differentiation, a single simple-appearing cyst on the right side, and a bifid right upper renal collecting system. Renal biopsy showed an irregularly increasing mesangial matrix with a moth-eaten appearance on silver staining, global sclerosis with tubular atrophy, and interstitial fibrosis. Immunofluorescence staining was positive only for complement component 3. Electron microscopy revealed glomerular capillary loops with membrane duplication, cellular interposition, and sub-endothelial and intramembranous deposits. The proband received a deceased-donor kidney transplant at the age of 9 years. The older sister of the proband also had overlapping congenital anomalies with bilateral iris and retinal coloboma, dysgenesis of the corpus callosum, bicornuate uterus, and mild anorectal problems that did not require surgery. In addition, she had hypoplastic left heart syndrome, and required a heart transplant at the age of 7 years (Figure 1A, Table 1). Older sister developed proteinuria at the age of 11 years and completed a 24 h urine collection which was noted to be elevated at 1590 mg/m^2^/day (nephrotic range > 1,000 mg/m^2^/day). However, her nephrologist stated that the sample might had been over collected as her creatinine excretion was 29 mg/kg (normal 18–20). At that time, the impression was that her persistent proteinuria was likely due to Sirolimus. Urine protein to creatinine ratio improved after stopping Sirolimus. Subsequently there was a negative urine analysis. She did not have a renal biopsy due to improvement of proteinuria.

**Table 1:** Phenotype of individuals with recessive *ADAMTS9* variants.

Individual	Nucleotide change	Amino acid change	Exon (Zygosity, Segregation)	Amino acid conservation in species	gnomAD allele frequencies	MT	CADD	SIFT	Gender	Ethnic origin	Renal manifestation	Renal biopsy	Extrarenal manifestations	References
-Proband -Older sister	c.1907C > T c.3072 T > A	p.S636F p.C1024^*^	Exon 13 (het, F) Exon 21 (het, M)	*D. rerio* N/A	ND ND	DC (1.00) N/A	29.4 35	Del (0) N/A	F F	Caucasian	Nephrotic range proteinuria, bilateral abnormal echogenicity, bifid right upper renal collecting system, bilateral single cyst, Simple 5 mm cyst in the midpole of the left kidney but otherwise normal kidney structure	Irregularly increased mesangial matrix with moth eaten appearance, global sclerosis with tubular atrophy and interstitial fibrosis, glomerular capillary loops with membrane duplication, cellular interposition, subendothelial and intramembranous deposit, and C3 glomerulonephritis	Hypertension, hydrocephaly, bicornuate uterus, anorectal abnormalities, bilateral retinal coloboma, absence or agenesis of corpus callosum, short stature, and tied tongue Hypoplastic left heart syndrome (s/p heart transplant at age 7 years), bilateral iris, retinal coloboma, agenesis of the corpus callosum, bicornuate uterus and vesicoureteral reflux	This study
F1279 -21*^a^*	c.4575_4576del	p.Gln1525Hisfs^*^60	Exon 30 (HOM)	N/A	ND	N/A	N/A	N/A	F	European	Increased echogenicity, medullary cysts, proteinuria ESKD at 5 years of age	ND	Cortical deafness, ASD growth retardation, coloboma, aplasia of vermis, and corpus callosum hypoplasia	(8)
A5048 -21*^a^*	c.194C > G	p.Thr65Arg	Exon 2 (HOM)	*C. elegans*	0.0002409 (1 homozygote)	DC (1.00)	23.5	Del (1.00)	M	Arabic	NPHP, nonselective proteinuria, and ESRD since infancy	Microcystic dilatation of tubules and immature glomeruli (at 2 years of age)	Sensorineural deafness, hepatosplenomegaly, short stature, anemia, thrombocytopenia, osteopenia, and rickets	(8)
-Proband	c.1940G > A c.3607C > T	p.Arg647Gln p.Arg1203Trp	Exon 13 (het, M) Exon 25 (het, F)	*X. tropicalis C. elegans*	0.0003931 (EA) 0.0001928 (EA)	DC (0.842) DC (1.00)	17.73 27.4	Del (1.00) 0.001	M	East Asian	ND	ND	Incomplete MTS, OMA, developmental delay, hypotonia, and mild bifid tongue.	(9)

ASD, atrial septal defect; CADD, Combined Annotation Dependent Depletion; DC, disease-causing; Del, deleterious; ESKD, end-stage kidney disease; F, female; gnomAD, Genome Aggregation Database; het, heterozygous; HOM, homozygous; M, male; MT, mutation taster; NA, not applicable; ND, no data; NPHP, nephronophthisis; OMA, oculomotor apraxia; MTS, molar tooth sign; SIFT, sorting intolerant from tolerant.

a These mutations have been reported previously.

ADAMTS9 cDNA mutations are numbered according to human cDNA reference sequence NM_182920.1.

To identify possible genetic causes, exome sequencing was performed on the proband. Biallelic mutations [c.1907C > T (chr3:64619505) and c.3072 T > A (chr3:64601114)] in *ADAMTS9* (NM_182920.2) were detected, which resulted in the production of missense (p.Ser636Phe) and nonsense (p.Cys1024*) ADAMTS9 protein variants, respectively. Segregation analysis using Sanger sequencing confirmed that her father was heterozygous for c.1907C > T, her mother was heterozygous for c.3072 T > A, and her older sister had both variants, indicating that the identified variants were segregated with the affected status in the family (Supplementary Figure S1A). The c.1907C > T variant has not been reported in the HGMD, ClinVar, and gnomAD databases. The serine at the 636th residue is evolutionarily conserved among vertebrates, suggesting that this residue may be essential for ADAMTS9 function (Figures 1B,C). In addition, multiple *in silico* tools predicted that this mutation could have deleterious effects on ADAMTS9 function: deleterious by SIFT with a score of 0, disease-causing by MutationTaster with a score of 1.00, and damaging by CADD with a score of 29.4 (Table 1). Additionally, the nonsense variant c.3072 T > A, has also not been reported in any database. This variant was predicted to be damaging by CADD with a score of 35.0 (Table 1).

Previously, clinical and genetic data of three patients with similar *ADAMTS9* variants have been reported (8, 9). In our study, kidney involvement was observed in three out of the five individuals with *ADAMTS9* variants. The older sister of this study had vesicoureteral reflux but did not have kidney disease and the other individual (c.1940G > A, p.Arg647Gln and c.3607C > T, p.Arg1203Trp) exhibited developmental delay and mild cerebellar vermis hypoplasia without NPHP or kidney dysfunction. Two of the three individuals with renal manifestations progressed to end-stage kidney disease (ESKD), while one individual showed ESKD since infancy. All individuals with renal manifestations had medullary cysts and exhibited proteinuria (Table 1), implying that ADAMTS9 is involved in regulating glomerular function.

### 3.2. Pathogenicity of *ADAMTS9* variants identified in this study

To examine the pathogenicity of the *ADAMTS9* variants (c.1907C > T; p.Ser636Phe and c.3072 T > A; p.Cys1024*), we first examined the effects of these mutations on ADAMTS9 expression and secretion (Supplementary Figure S2A). The expression levels of wild-type and mutant ADAMTS9 in cells were comparable, suggesting that these mutations had no effect on the expression or intracellular stability of this protein. Next, we examined the ADAMTS9 protein levels in the culture media. No difference was observed between mutant and wild-type ADAMTS9 levels in the culture media, implying that these mutations had no effect on ADAMTS9 secretion (Supplementary Figure S2A). Finally, we examined the intracellular localization of wild-type and mutant ADAMTS9 proteins. ADAMTS9 is secreted into the extracellular space, following which the protein is endocytosed and re-localized near the basal body of primary cilia (8, 10). RPE1 cells were transfected with expression plasmids carrying C-terminal V5-tagged wild-type or mutant ADAMTS9. Cells were serum starved and the localization of ADAMTS9 was observed by staining with the anti-V5 antibody. Wild-type ADAMTS9 was localized near the base of primary cilia, whereas the p.Cys1024* mutant showed no such localization (Figure 1D). For the p.Ser636Phe mutant, localization was observed only in a few transfected cells (approximately 30%) with primary cilia. These results indicate that the mutant *ADAMTS9* variants identified in our study have impaired sub-cellular localization, which is similar to previously identified *ADAMTS9* variants.

### 3.3. Modeling ADAMTS9-related nephropathy using kidney organoids

**Figure 2:**
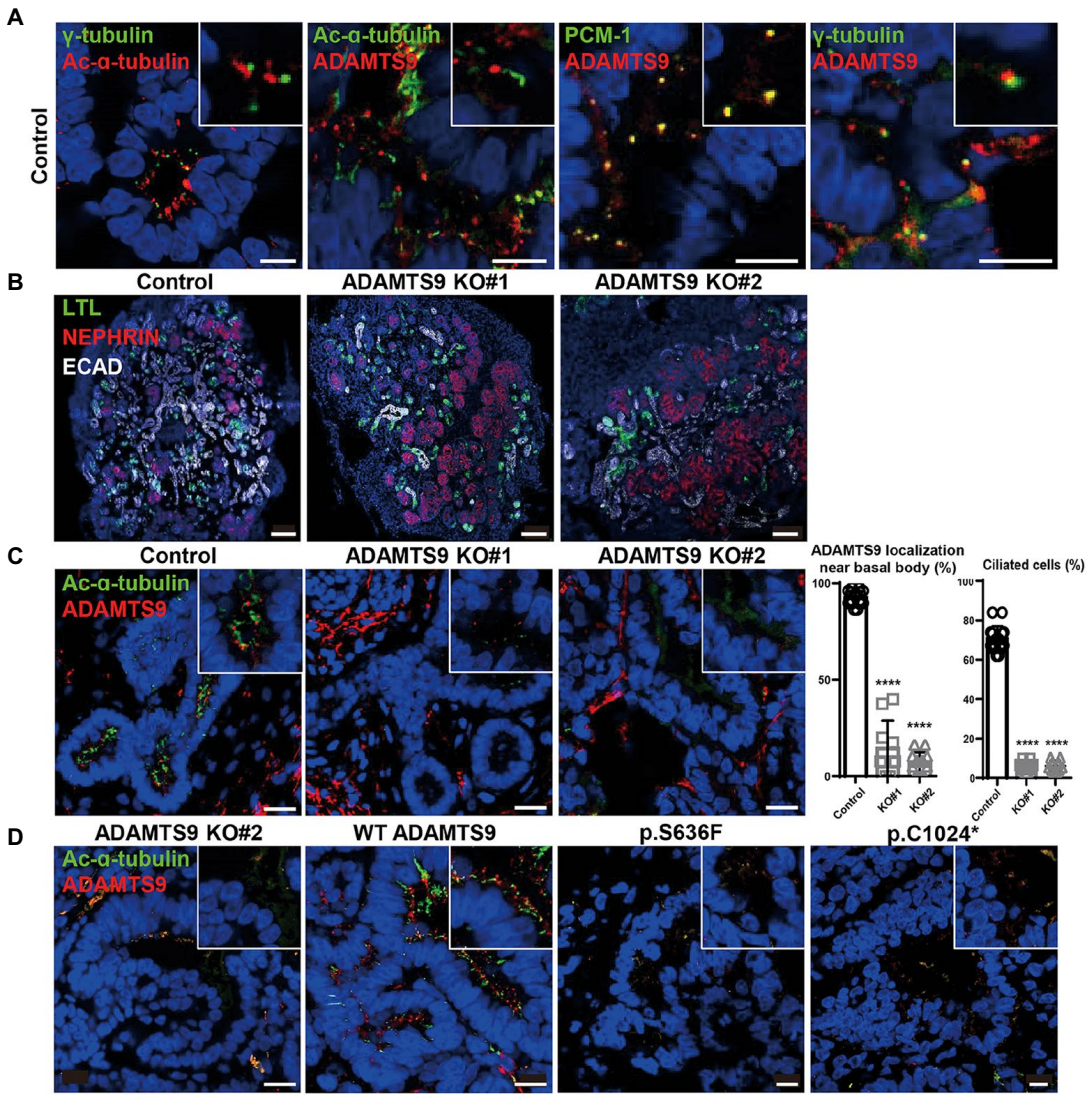
*ADAMTS9* knockout kidney organoids demonstrate abnormal cilia, and ciliogenesis is not rescued by overexpressing ADAMTS9 variants. **(A)** ADAMTS9 is localized near the basal bodies of primary cilia and co-localized with PCM-1. The organoids were stained with anti-ADAMTS9, anti-Ac-α-tubulin, anti-PCM1, or anti-γ-tubulin antibodies. Scale bar, 10 μm. **(B)** Immunofluorescence staining of the kidney organoids displaying different structures, including the glomerulus (NEPHRIN), proximal tubules (LTL), and distal tubules (ECAD). Scale bar, 100 μm. **(C)** Loss or shortening of primary cilia was observed in *ADAMTS9* knockout organoids. Percentage of ciliated tubular cells in kidney organoids and quantification of cilium length on the basis of Ac-α-tubulin staining are shown in the right panel. Data represent means ± standard deviation (SD). Scale bar, 10 μm. ^****^p < 0.0001. **(D)** Ciliogenesis was rescued by overexpressing wild-type but not by mutant forms of ADAMTS9, in *ADAMTS9*-ablated kidney organoids. Scale bar, 10 μm.

To investigate the role of ADAMTS9 in the kidney, we generated kidney organoids that contain glomeruli and tubules. Kidney organoids were generated from hiPSCs which were induced toward embryonic nephron progenitor cells (11). The embryonic nephron progenitos were confirmed by existence of ITGA8-positive fractions (Supplementary Figure S4) and were cultured for another 29 days for nephrogenesis. ADAMTS9 localization was then examined in cells with primary cilia. Co-staining with acetylated-α-tubulin, PCM-1, and γ-tubulin confirmed that ADAMTS9 localizes near the basal bodies of primary cilia in the cells of kidney organoids (Figure 2A), which is in agreement with previous studies on cell lines (8, 10). Next, we generated *ADAMTS9* knockout hiPSCs using CRISPR/Cas9 and confirmed that both alleles were inactivated using Sanger sequencing (Supplementary Figure S3A). The control and ADAMTS9 knockout organoids were positive for NPHS1 (a podocyte marker), LTL (a proximal tubule marker), and ECAD (a distal tubule marker) expression, with no difference between control- and *ADAMTS9* knockout hiPSC-derived kidney organoids (Figure 2B, Supplementary Figures S5A,B), implying that *ADAMTS9* ablation does not affect nephron development.

Although tubulogenesis was not affected, it was difficult to observe the ciliated cells in knockout organoids (Figure 2C). To confirm whether this ciliary defect is due to the loss of ADAMTS9, we performed rescue experiments in kidney organoids. The overexpression of wild-type ADAMTS9 rescued ciliogenesis, whereas the overexpression of mutant *ADAMTS9* variants failed to rescue ciliogenesis (Figure 2D), confirming the role of ADAMTS9 in primary cilia formation and pathogenicity of the identified variants. Moreover, our data showed that kidney organoids derived from *ADAMTS9* knockout hiPSCs recapitulate ciliary defects as observed in NPHP-RC.

### 3.4. Single-cell transcriptomics of *ADAMTS9* knockout hiPSC-derived kidney organoids revealed the role of ADAMTS9 in podocytes

**Figure 3:**
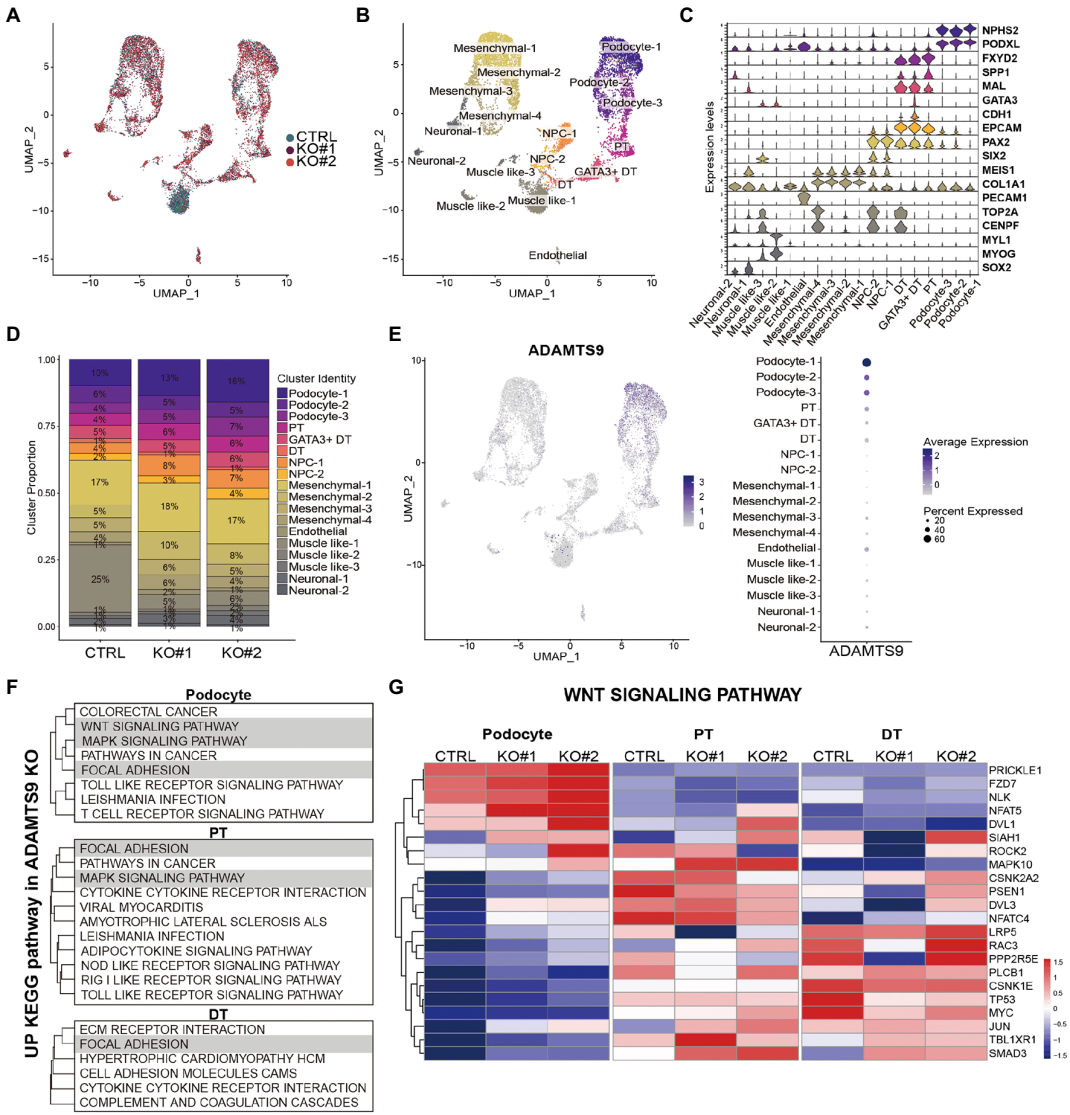
scRNA-seq analysis of the control and *ADAMTS9* knockout kidney organoids. **(A)** UMAP visualization of single-cell transcriptomic profiles from two *ADAMTS9* knockout organoids and a control organoid. **(B)** UMAP plots showing 18 clusters identified by canonical marker genes. **(C)** Proportion of clusters across control and *ADAMTS9* knockout organoids with corresponding percentages. **(D)** Violin plots showing the expression of canonical and data-derived kidney markers used to identify nephron clusters in D29 organoids: podocytes (NPHS2, PODXL), proximal tubules (FXYD2, SPP1), distal tubules (MAL, CDH1), immature distal nephrons (GATA3 + DT), and nephron progenitor cells (PAX2, SIX2). Mesenchymal, endothelial, and off-target cells were also identified. **(E)** ADAMTS9 expression across all clusters in control organoids. **(F)** Analysis of leading edge genes from GSEA of differentially expressed genes in podocytes, PT, and DT clusters showed three enriched pathways: focal adhesion, MAPK pathway, and Wnt pathway. **(G)** Heatmap showing expression of Wnt signaling pathway-related genes identified by GSEA in podocytes, PT, and DT clusters in control versus *ADAMTS9* knockout kidney organoids.

To uncover the gene expression profiles of various cell types present in the kidney organoids at single-cell resolution, single-cell RNA sequencing was performed using the 10x Genomics Chromium platform. Experiments were performed twice and data from each cell type were combined. Overall, 12,093 cells were profiled (Supplementary Figures S6A–C). Uniform manifold approximation and projections (UMAPs) comparing cells from *ADAMTS9* knockout versus control samples revealed a high degree of overlap (Figure 3A). Using cell-type specific gene signatures (12), we identified all nephron cell types, including podocytes, tubular cells, and progenitor cells, in addition to mesenchymal cells, endothelial cells, and off-target populations. These cells were classified into 18 clusters based on their canonical markers (Figures 3B,C). The clusters were identified in both control as well as *ADAMTS9* knockout organoids (Figure 3D).

Since kidney organoids exhibit heterogeneity at the cellular levels, we used CellphoneDB to examine the cell–cell interaction networks among different cell types present in the organoids. Most interactions, including WNT, BMP, VEGFA, and SHH interactions, which are critical for the development of kidney organoids, were not affected by ADAMTS9 deficiency. The only exception was TGF-β1, which is expressed in podocytes and showed increased interaction with endothelial TGFBR1 and TGFBR2, in *ADAMTS9* knockout organoids (Supplementary Figure S7A). To verify the role of ADAMTS9 in regulating kidney organoid maturation and developmental transitions, we conducted pseudo-time analysis using Monocle3. Gene expression along the pseudo-time trajectory showed the expected segregation of markers that were not affected by *ADAMTS9* ablation (Supplementary Figures S7B,C).

We observed that the number of cells in the podocyte cluster was higher in knockout organoids, although the total number of cells in knockout organoids was relatively lower (Figure 3D, Supplementary Figure S6C). In control organoids, most ADAMTS9-expressing cells were podocytes, followed by proximal tubule cells (Figure 3E). To confirm the expression pattern of ADAMTS9 in podocytes, we looked into the openly available Human Protein Atlas (HPA)^2^ (13). ADAMTS9 is abundantly expressed in podocytes and endothelial cells of glomeruli (Supplementary Figure S8A), which is consistent with the results of ADAMTS9 expression of scRNA-seq data. Overall, single-cell RNA sequencing analysis confirmed that cells in the ADAMTS9 knockout organoids are of the nephron lineage, and that ADAMTS9 is abundantly expressed in the podocyte cluster, which may explain the glomerular proteinuria observed in individuals with recessive *ADAMTS9* mutations. To better understand the mechanisms leading to proteinuria in individuals with ADAMTS9 dysfunction, we compared the gene expression profiles of podocyte and tubular clusters derived from control and knockout organoids. Gene set enrichment analysis (GSEA) revealed that genes regulating the MAPK signaling pathway, focal adhesion, and Wnt signaling pathway (Figure 3F, Supplementary Figures 9A,B, Supplementary Table S1) were enriched in podocytes derived from knockout organoids. The MAPK signaling pathway integrates cell stimuli to regulate several cellular functions (14). Since ADAMTS9 is a protease, it is speculated that this enzyme is also associated with the regulation of focal adhesion (15). Moreover, the Wnt signaling pathway is a central signaling pathway that regulates cell migration, polarity, and patterning during early embryonic development (16).

### 3.5. Localization of nephrin and podocin is impaired in *ADAMTS9* knockout kidney organoids

**Figure 4:**
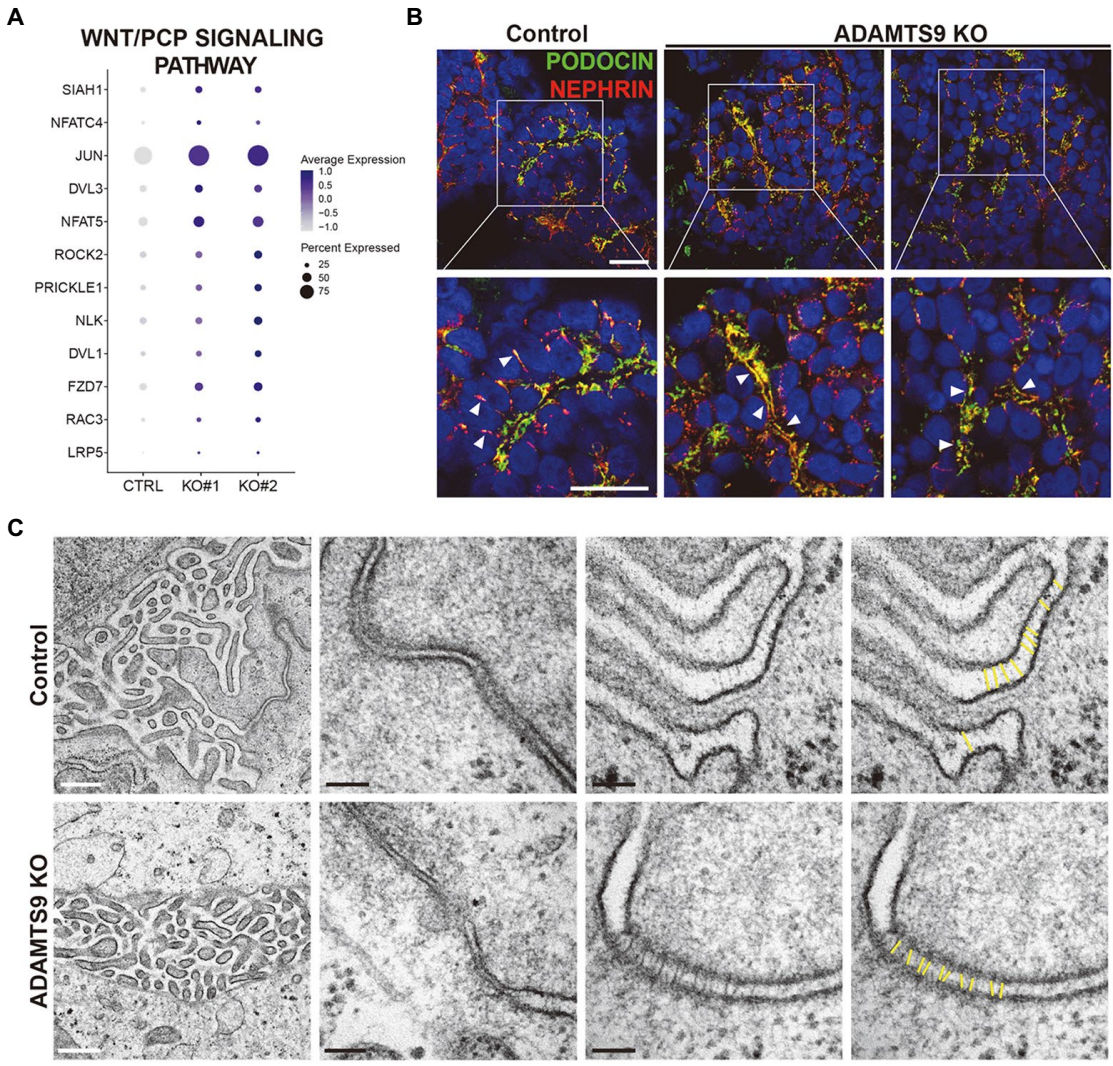
ADAMTS9 influences the Wnt/PCP pathway and regulates the localization of junction proteins. **(A)** Dot plot showing the expression of Wnt/PCP pathway genes in podocyte clusters across control and *ADAMTS9* knockout (KO) organoids. **(B)** Immunostaining of kidney organoids showing NEPHRIN and PODOCIN localization in *ADAMTS9* KO podocytes. White arrows indicate NEPHRIN and PODOCIN localization. White arrows indicate NEPHRIN and PODOCIN localization. NEPHRIN and PODOCIN were observed in the lateral side of podocytes in control organoids, but not in *ADAMTS9* KO organoids. NEPHRIN and PODOCIN were localized only in the basal side of podocytes in *ADAMTS9* KO organoids. Scale bars, 10 μm. **(C)** Electron microscopy images of podocytes in kidney organoids. Ladder-like structures were observed in both control and *ADAMTS9* KO podocytes. White scale bars, 500 nm. Black scale bars, 100 nm.

We observed that the Wnt signaling pathway is perturbed in podocytes from *ADAMTS9* knockout organoids (Figure 3G). Moreover, GSEA results revealed that genes enriched in knockout podocytes were associated with the β-catenin-independent or non-canonical Wnt signaling pathway (Supplementary Figures 10A,B), also known as the planar cell polarity (PCP) pathway (Figure 4A). The PCP pathway affects podocyte development by regulating nephrin turnover during junctional remodeling as cells differentiate (17). We examined the localization of junctional proteins in podocytes by immunostaining. Results showed that nephrin and podocin were localized to the lateral and basal regions of podocytes in control organoids (Figure 4B). However, in *ADAMTS9* knockout podocytes, both nephrin and podocin were undetectable on the lateral side and their expression was located to the basal side, which implied the matured status of podocytes (Figure 4B). Moreover, electron microscopy of *ADAMTS9* knockout podocytes showed foot processes and filtration slits that were connected with ladder-like structures as did the control organoids (Figure 4C). Although *ADAMTS9* knockout podocytes also formed foot processes with ladder-like junctional structures, intercellular junctions showed tighter adherence than control cells. The processes of *ADAMTS9* knockout podocytes were closely localized in adjacent cells. The intercellular space is associated with junctional protein complex which maintaining epithelial cell polarity (18–20). Taken together, these results indicate that ADAMTS9 regulates the Wnt/PCP signaling pathway, without affecting slit diaphragm-associated proteins in podocytes.

## 4. Discussion

In this study, we identified novel compound heterozygous variants of *ADAMTS9* and investigated the role of these variants in an effort to discover disease-specific pathways. Homozygous frameshift variants (p.Gln1525Hisfs*60) of *ADAMTS9* have been associated with severe brain anomalies and renal dysfunction (8), whereas compound heterozygous mutations (p.Arg647Gln and p.Arg1203Trp) have been observed in Joubert syndrome-related disorders without any renal dysfunction (9). Herein, we identified novel compound heterozygous variants of *ADAMTS9* in a family with multiple congenital birth defects. Renal biopsy of a proband with *ADAMTS9* variants (p.Ser636Phe and p.Cys1024*) revealed renal interstitial fibrosis with an abnormal thickness of the mesangial matrix accompanied by unusual proteinuria. Retrospectively, we hypothesize that older sister’s nephrotic range proteinuria might have been due to her variants in *ADAMTS9* exacerbated by her exposure to Sirolimus. Sister did not have an indication for renal biopsy, hence further information about her renal pathological findings are not available.

Although previous studies investigating ADAMTS9 functions relied on animal models or immortalized cells (10, 21–23), these models were unable to recapitulate specific dysfunctions and phenotypes observed in affected individuals. Thus, it is necessary to model renal diseases using human tissues. Recently, advances have been made toward the directed differentiation of iPSCs into renal lineages, including podocytes and tubular segments (2). Although modeling NPHP using kidney organoids produced from iPSCs derived from individuals with inherited *IFT140* variants has been reported (24), the present study demonstrated the role of NPHP-associated genes in glomerular diseases and the importance of kidney organoids in association with transcriptomic analysis.

In agreement with previous reports of abnormal ciliary structures in NPHP models, we observed that the newly identified mutations in *ADAMTS9* resulted in the shortening or loss of primary cilium. Furthermore, all identified ADAMTS9 mutant proteins failed to localize near the basal body of primary cilia, suggesting that this ciliary localization is essential for ADAMTS9 function.

We noted that TGF-β signaling pathway was the only signaling that showed alteration in cell–cell interaction between podocytes and endothelial cells upon loss of *ADAMTS9* (Supplementary Figure S7A). TGF-β has long been considered as a key mediator of renal fibrosis. GSEA on scRNA-seq data from *ADAMTS9* knockout organoids compared with control (Supplementary Table S1) revealed TGF-β signaling as positively enriched pathway in podocytes, implicating renal inflammation and fibrosis. Recently, TGF-β induced endothelial to mesenchymal transition (25, 26) which also has been implicated in fibrosis. These data indicate that crosstalk between podocytes and endothelial cells regulates extracellular accumulation. However, our kidney organoids were hampered to fully develop into functional kidneys as evidenced by the lack of functional vascularization and absence of stromal progenitors. In addition, ADAMTS9 regulates vascular development in the umbilical cord and PDGFRβ signaling *via* MAPK activation (23). Although our scRNA-seq data revealed that MAPK signaling is altered in epithelial clusters, the resulting fibrosis signal was not detected; thus, the precise functional role of ADAMTS9 in the kidney remains elusive.

Our single-cell transcriptomic analysis demonstrated that ADAMTS9 is highly expressed in the podocyte cluster and identified that the Wnt/PCP signaling pathway is perturbed in the *ADAMTS9* knockout cluster. The PCP pathway is a key conserved pathway in epithelial and mesenchymal cells that plays an important role during development (27, 28). It is well accepted that primary cilia play an important role in the PCP signaling pathway (24, 29, 30). Normal ciliogenesis aids in the establishment of cell polarity, which is dependent on the migration of the basal body to the apical cell surface to define apicobasal polarity (30). In addition, a previous study demonstrated that ADAMTS9 depletion resulted in mislocalization of β-catenin at basolateral membrane and loss of apicobasal polarity using spheroid model of IMCD3 cells (8), suggesting that ADAMTS9 is responsible for cellular polarity in tubular cells. Although our results failed to sufficiently explain how ADAMTS9 regulates the PCP pathway in podocytes, a previous study showed the association between the PCP pathway and metalloprotease trafficking. MMP14, a membrane type-1 matrix metalloproteinase, is recruited downstream of Vangl2, a PCP pathway protein. This temporal recruitment of MMP14 leads to the proteolytic degradation and remodeling of the ECM (31, 32). Since ADAMTS9 is speculated to have proteolytic activity intracellularly that plays an important role in ciliogenesis (10), further studies are needed to confirm that the proteolytic activity of ADAMTS9 is necessary for the development of glomerular diseases.

PCP also has profound effect on podocyte shape, actin rearrangement and junctional protein complexes and associated with podocyte-related diseases (9, 17). VANGL2 transcripts levels were increased in the glomeruli of individuals with focal segmental glomerulosclerosis (33). Activation of the PCP pathway stimulates nephrin endocytosis in cultured podocytes (17). Several kidney diseases in both children and adults begin with proteinuria, and abnormal nephrin localization and SD formation are implicated in these glomerular diseases (34). However, our data revealed that nephrin localization was sustained in ADAMTS9-depleted organoids. These results suggest that expression of junctional proteins is independent of PCP signaling which regulated by ADAMTS9 in podocytes.

Taken together, our data provide insights into the pathogenic effects of ADAMTS9 dysfunction and identified a novel role of ADAMTS9 in glomeruli.

## Data availability statement

The datasets presented in this study can be found in online repositories. The names of the repository/repositories and accession number(s) can be found at: https://www.kobic.re.kr/kona, PRJKA220508.

## Ethics statement

The studies involving human participants were reviewed and approved by Baylor College of Medicine. Written informed consent to participate in this study was provided by the participants’ legal guardian/next of kin.

## Author contributions

MRB performed genetic evaluation and conceived the study. NB recruited the family and gathered the clinical information. HD and SLG performed exome sequencing and segregation analysis. SY, YJC, H-YK, JHR, SL, and RN performed molecular and organoid experiments. SY and FH performed the single-cell RNA sequencing and analyzed the data. MRB and HYG implemented the entire project and wrote the paper with help from SY. All authors contributed to the article and approved the submitted version.

## Funding

HYG was supported by the National Research Foundation of Korea (NRF) funded by the Korean government (MSIT, 2018R1A5A2025079).

## Conflict of interest

The authors declare that the research was conducted in the absence of any commercial or financial relationships that could be construed as a potential conflict of interest.

## Publisher’s note

All claims expressed in this article are solely those of the authors and do not necessarily represent those of their affiliated organizations, or those of the publisher, the editors and the reviewers. Any product that may be evaluated in this article, or claim that may be made by its manufacturer, is not guaranteed or endorsed by the publisher.

## Footnotes

^1^ https://github.com/zktuong/ktplots

^2^ http://www.proteinatlas.org
