# Supplementary material for: Disease modeling of ADAMTS9-related nephropathy using kidney organoids reveals its roles in tubular cells and podocytes
Source: Front Med (Lausanne). 2023 Mar 23;10:1089159. doi: 10.3389/fmed.2023.1089159 (PMC10079903; doi:10.3389/fmed.2023.1089159)
Supplement: Supplementary file 1 [file Data_Sheet_1.PDF]

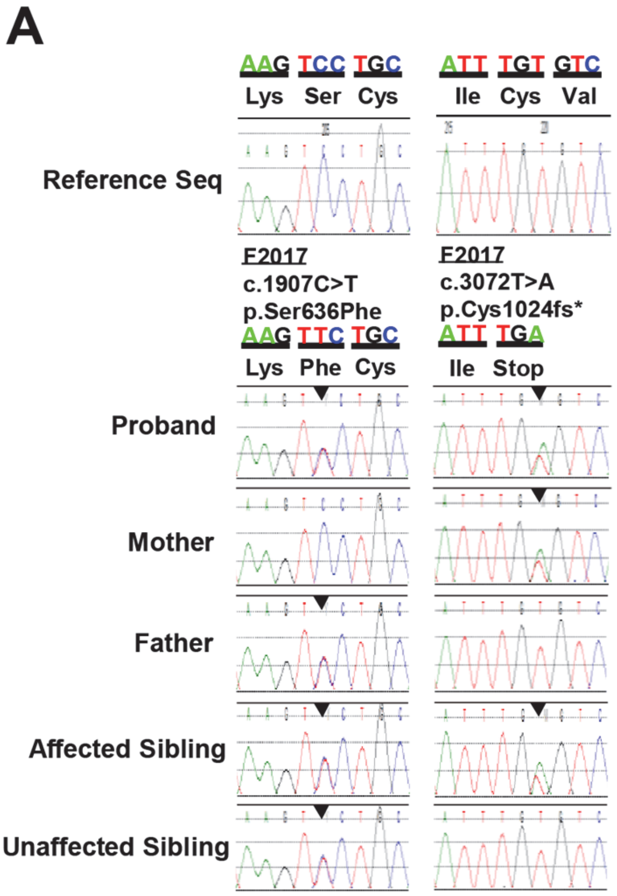

Supplementary Figure 1. Detection of causative variants in *ADAMTS9*.
(A) Sanger sequencing traces of *ADAMTS9* variants. Altered nucleotides (arrowheads) and amino-acid changes are given above sequence traces. Wild-type reference sequences are shown above sequences of the family. Codon triplets are underlined to indicate the reading frame.

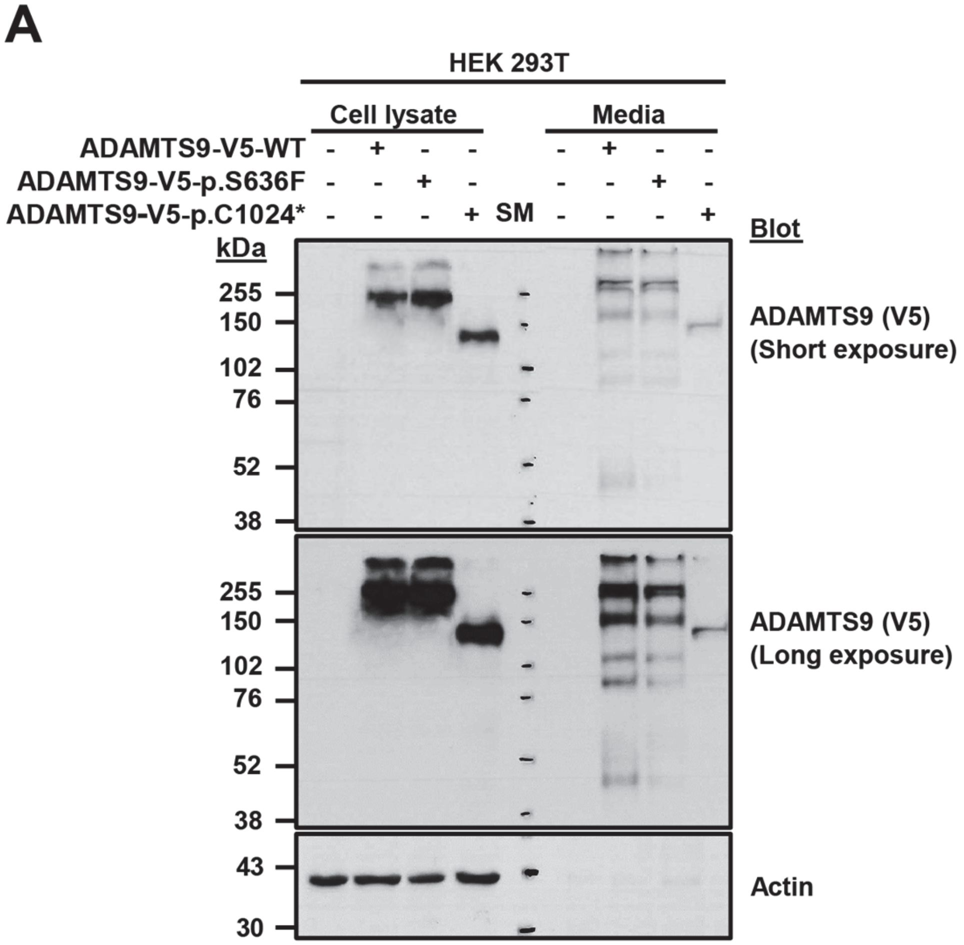

Supplementary Figure 2. The effect of *ADAMTS9* variants on protein secretion.

(A) Wild-type and mutant *ADMATS9* were transfected into HEK 293 cells and secreted ADAMTS9 was collected from culture media. Both mutant proteins as well as wild-type ADAMTS9 protein were detected in the culture media of transfected cells, indicating that the secretion of ADAMTS9 is not affected by the mutations.

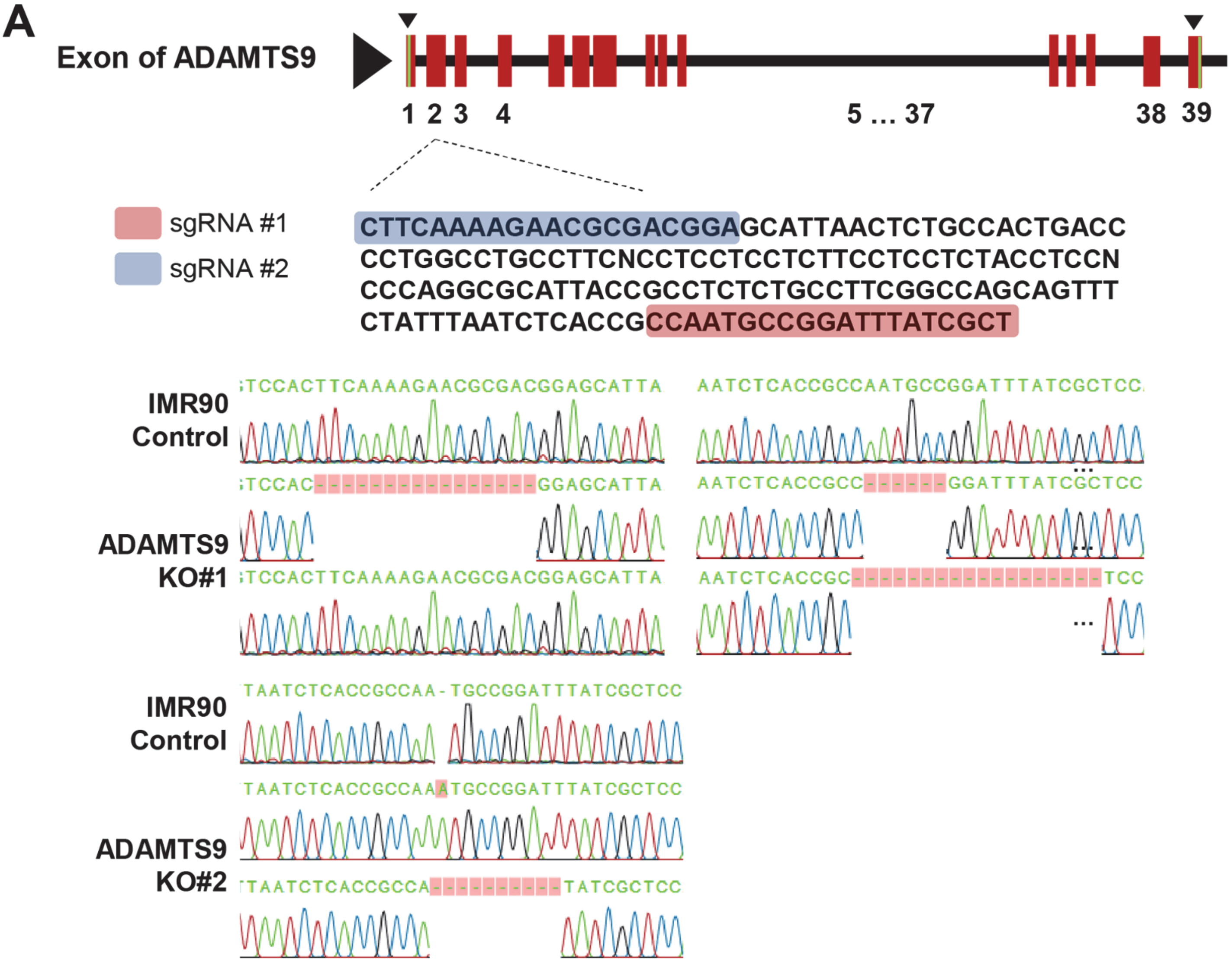

Supplementary Figure 3. Generation of ADAMTS9 knockout iPSCs.

(A) Two sgRNAs targeting the exon 2 of ADAMTS9 were designed. The sequence of target genomic DNA region is shown in the upper panel. Sanger sequencing of the exon 2 of ADAMTS9 confirmed genome editing in knockout (KO) cells (lower panel).

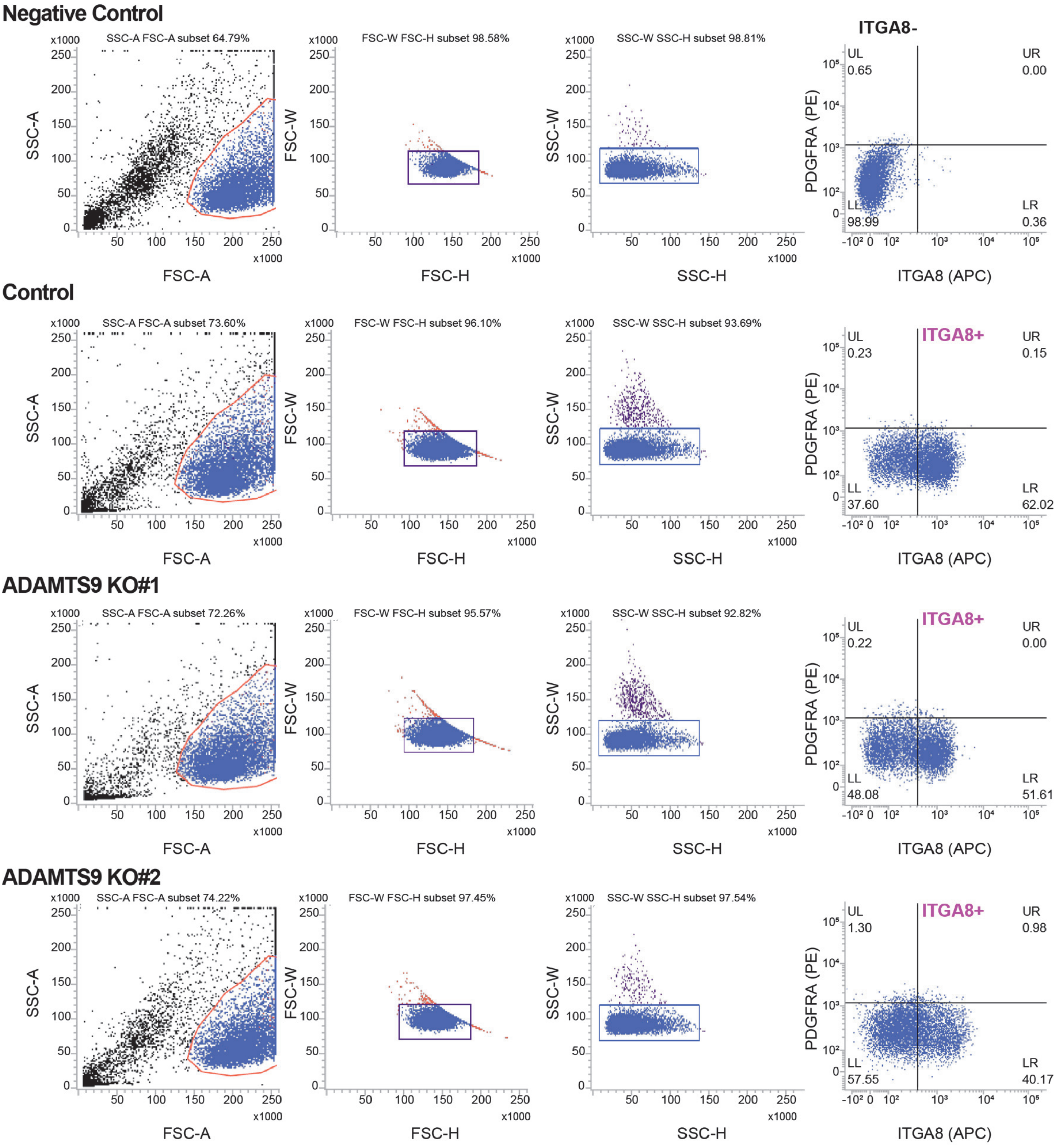

Supplementary Figure 4. FACS sequential gating/sorting strategies.
Dissociated cells were directly stained with PE-labeled PDGFRA and APC-labeled ITGA8 and analyzed by flow cytometry. Live cells were initially gated with the FSC/SSC and then FSC-H/FSC-W was applied to exclude doublet cells. The isolated cell populations were subsequently gated with embryonic nephron progenitor marker (ITGA8+) as indicated in the figures.

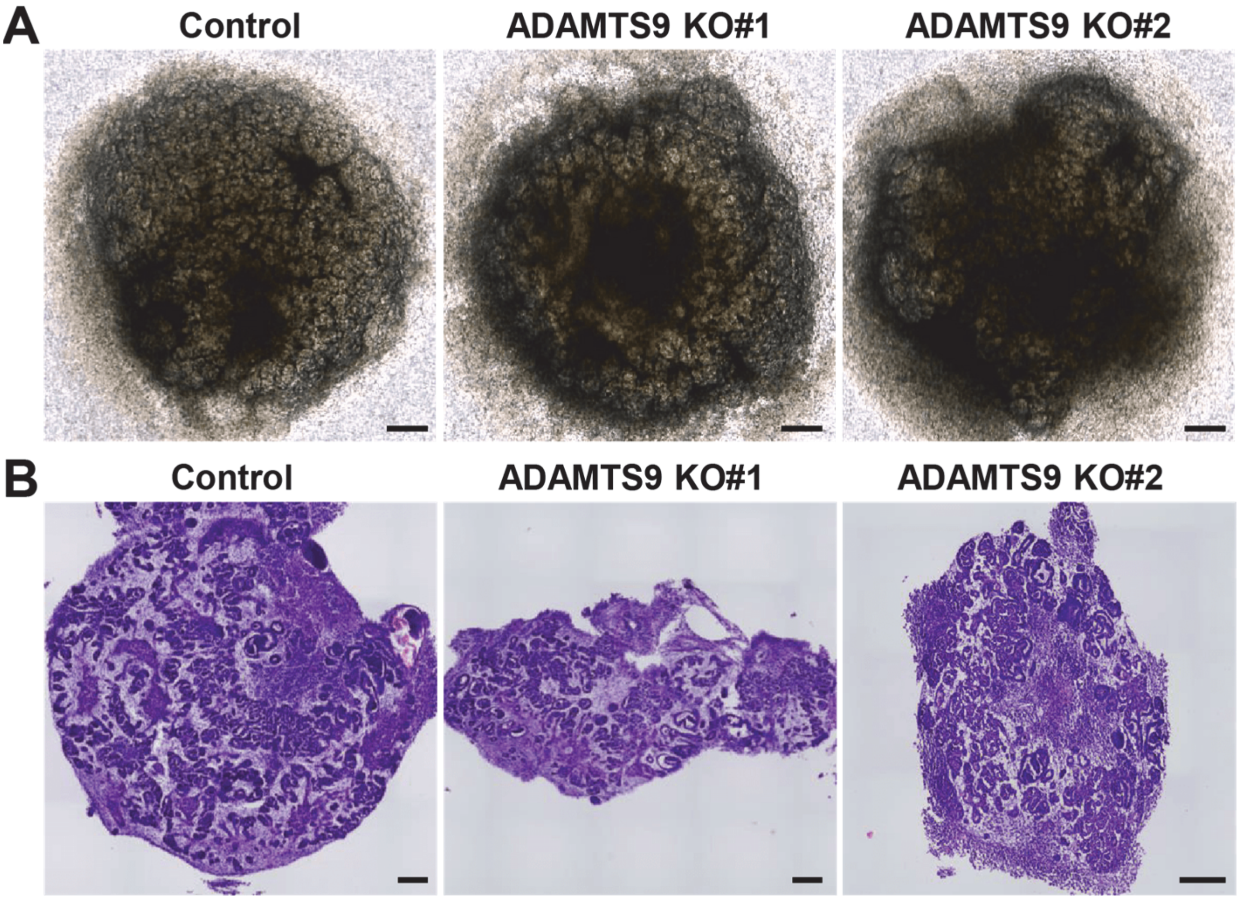

Supplementary Figure 5. Representative images showing morphology of kidney organoids and ADAMTS9 expression in kidney tissue.

(A) Bright-field images of control and ADAMTS9 knockout (KO) organoids at 7 days after induction of nephron progenitor cells. Scale bar, 100 μm.

(B) Hematoxylin & eosin staining of -control and ADAMTS9 KO organoids. Nephron progenitor cells showed three-dimensional nephron structures when culture in basal media for 29 days. Scale bar, 100 μm.

A

	Batch #	Median reads / Cell	Cells	After filter		Mean (nCount)	Mean (nFeature)
CTRL1	1	31977	7096	1504	5561	13447	3947
CTRL2	2	31385	10304	4057		6536	2208
KO#1	1	15516	5704	862	3372	7944	2912
KO#1	2	22413	5483	2510		10343	3188
KO#2	1	17348	6521	971	3160	8527	3073
KO#2	2	22843	6285	2189		10603	3323

B

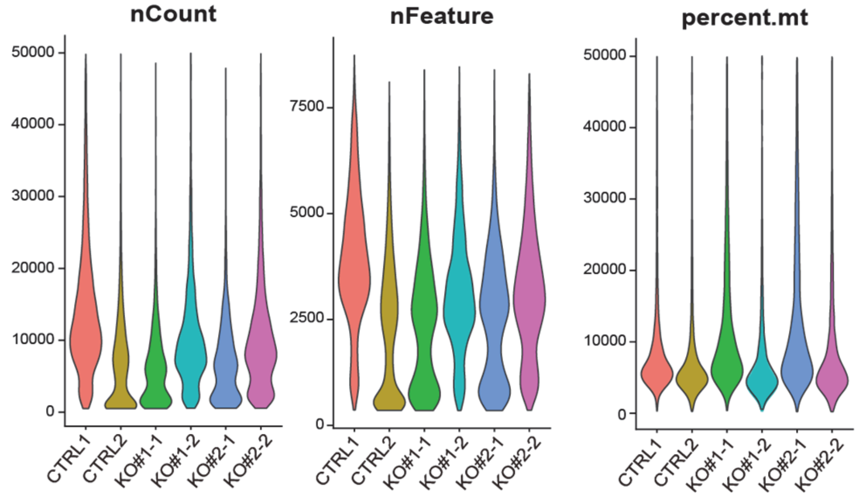

C

	CTRL	KO#1	KO#2
Podocyte-1	540	454	504
Podocyte-2	361	175	173
Podocyte-3	217	177	230
PT	248	205	185
GATA3+ DT	277	154	178
DT	83	39	30
NPC-1	228	263	223
NPC-2	138	91	127
Mesenchymal-1	930	614	531
Mesenchymal-2	271	353	238
Mesenchymal-3	298	191	149
Mesenchymal-4	215	186	133
Endothelial	51	66	34
Muscle like-1	1405	181	174
Muscle like-2	70	34	64
Muscle like-3	62	29	51
Neuronal-1	124	117	112
Neuronal-2	43	43	24
Total :	5561	3372	3160

Supplementary Figure 6. Single-cell RNA sequencing of control and ADAMTS9 knockout kidney organoids.

(A,B) Quality control measures of single-cell RNA sequencing (scRNA-seq) for organoids samples.

(C) Proportions of clusters by samples (left) with corresponding cell numbers (right).

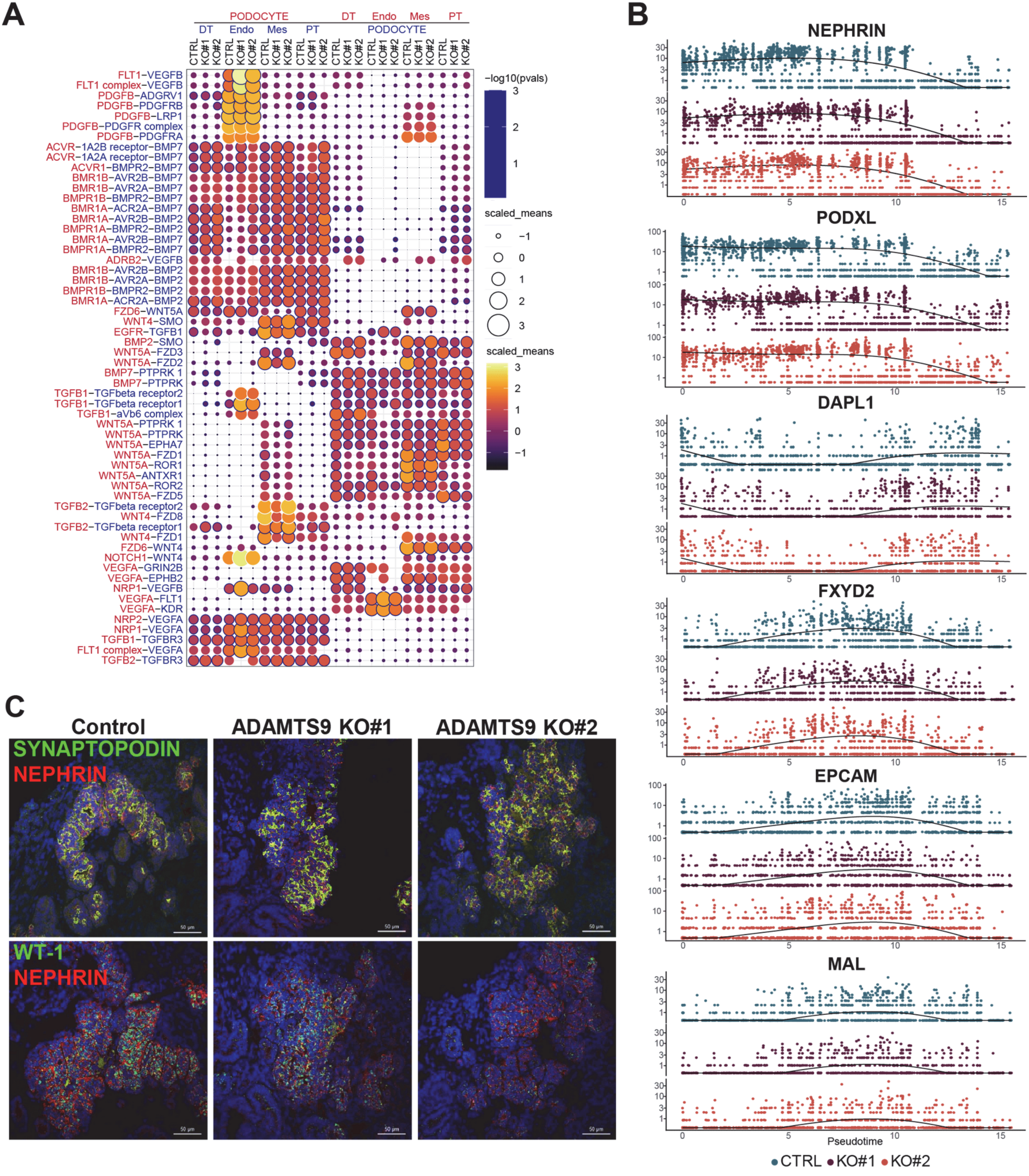

Supplementary Figure 7. Cell communication network and differentiation status in control and ADAMTS9 knockout kidney organoids.

(A) Bubble plot showing the selected ligand-receptor interactions with podocytes. Scaled means indicated by color and p-value by circle size.

(B) Expression of representative nephron marker genes across the pseudotime trajectory colored by Monocle state. ADAMTS9 knockout (KO) did not affect the expression of kidney markers in pseudotime state.

(C) Immunofluorescence of nephron organoids displaying podocyte markers including WT1, NEPHRIN, and SYNAPTOPODIN. Expressions of podocyte markers were not different between control and ADAMTS9 KO organoids. Scale bar, 100 μm.

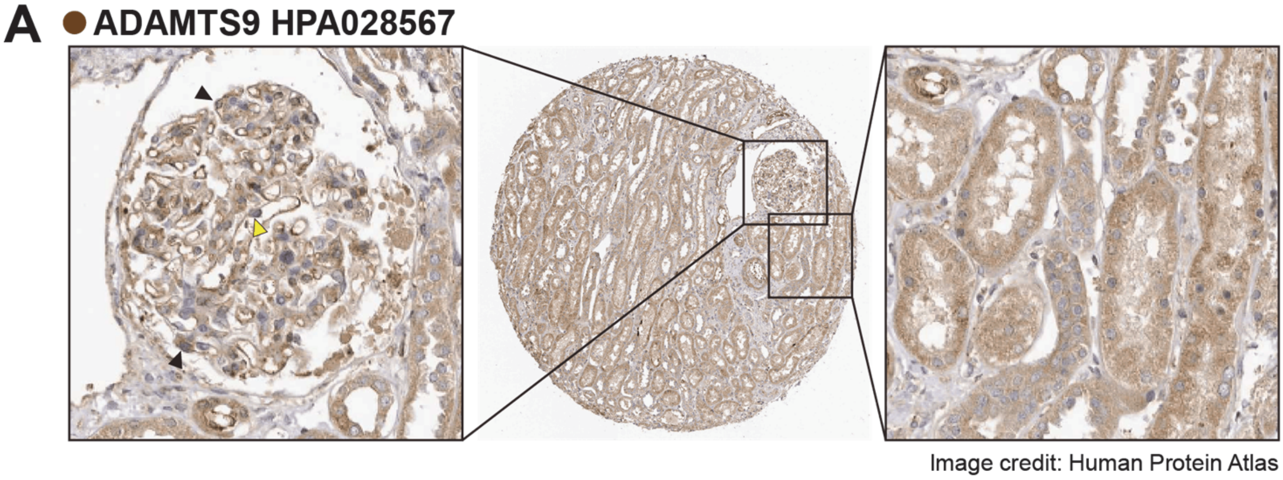

Supplementary Figure 8. Expression of ADAMTS9 in human kidney.
(A) ADAMTS9 expression of normal kidney tissue in the Human Protein Atlas. ADAMTS9 was detected in podocyte (black arrowhead) and endothelial cells (yellow arrowhead) of glomeruli.

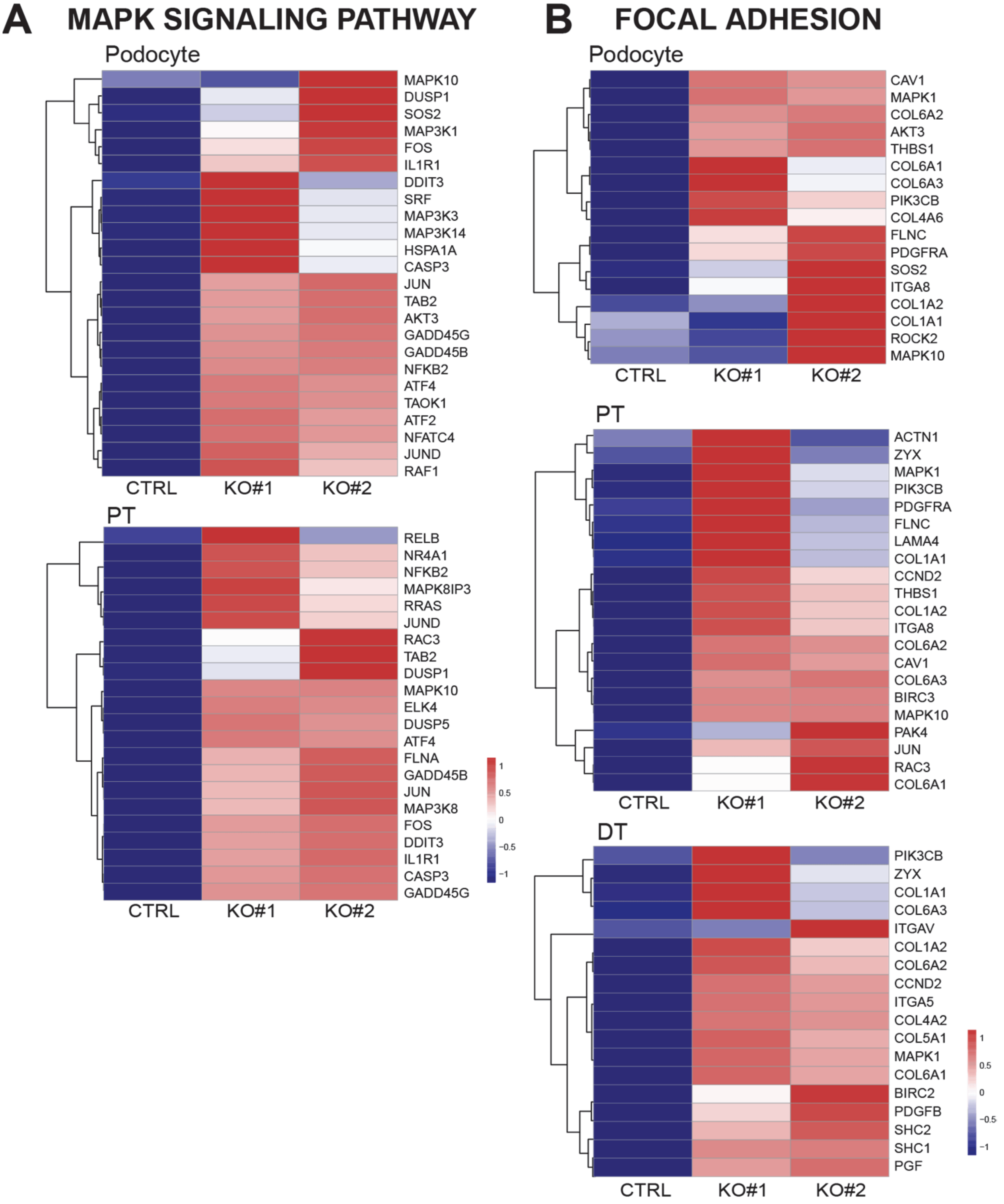

Supplementary Figure 9. Genes related to focal adhesion and MAPK are upregulated in ADAMTS9 knockout kidney organoids.

(A-B) Heatmap of genes related to MAPK pathway (A) and focal adhesion (B) in podocyte, proximal tubule (PT), and distal tubule (DT) clusters in control and ADAMTS9 knockout (KO) kidney organoids. Gene sets are from Gene Set Enrichment Analysis (GSEA).

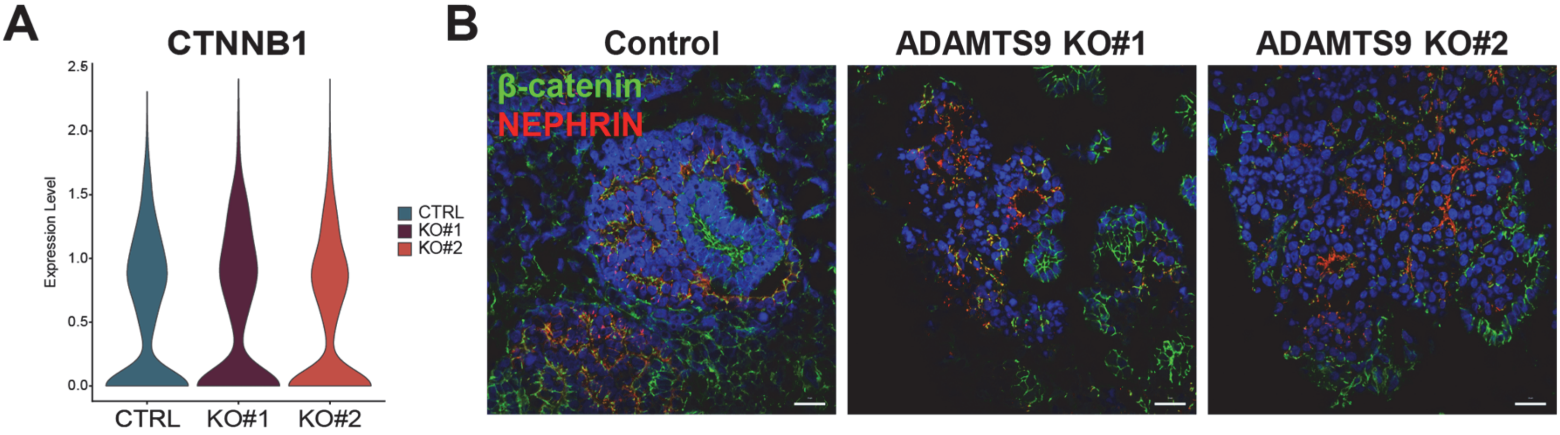

Supplementary Figure 10. Expression of β-catenin in control and ADAMTS9 knockout kidney organoids

(A) Violin plot of β-catenin (CTNNB1) expression across control and ADAMTS9 knockout (KO) kidney organoids.

(B) Immunofluorescence staining for β-catenin and NEPHRIN in control and ADAMTS9 KO organoids. ADAMTS9 deficiency did not affect the expression of β-catenin. Scale bars, 20 μm.
